# Polymer Scaffolds-Enhanced Bone Regeneration in Osteonecrosis Therapy

**DOI:** 10.3389/fbioe.2021.761302

**Published:** 2021-09-24

**Authors:** Hengliang Dong, Tongtong Zhu, Mingran Zhang, Dapeng Wang, Xukai Wang, Guanning Huang, Shuaishuai Wang, Minglei Zhang

**Affiliations:** ^1^ Department of Orthopedics, China-Japan Union Hospital of Jilin University, Changchun, China; ^2^ Department of Orthopedics, Siping Central Hospital, Siping, China

**Keywords:** polymer scaffold, bone tissue engineering, osteonecrosis treatment, bone regeneration, functionalization

## Abstract

Osteonecrosis without effective early treatment eventually leads to the collapse of the articular surface and causes arthritis. For the early stages of osteonecrosis, core decompression combined with bone grafting, is a procedure worthy of attention and clinical trial. And the study of bone graft substitutes has become a hot topic in the area of osteonecrosis research. In recent years, polymers have received more attention than other materials due to their excellent performance. However, because of the harsh microenvironment in osteonecrosis, pure polymers may not meet the stringent requirements of osteonecrosis research. The combined application of polymers and various other substances makes up for the shortcomings of polymers, and to meet a broad range of requirements for application in osteonecrosis therapy. This review focuses on various applying polymers in osteonecrosis therapy, then discusses the development of biofunctionalized composite polymers based on the polymers combined with different bioactive substances. At the end, we discuss their prospects for translation to clinical practice.

## Introduction

Osteonecrosis is a disease caused by a temporary or permanent loss of blood supply to the bone. The causes of osteonecrosis are extensive, and the pathogenesis is still unclear (Guo et al., 2014; Cui et al., 2021; Hines et al., 2021). The potential pathogenic factors that have been explored include trauma (Pascarella et al., 2019), long-term history of heavy drinking (Chen et al., 2017), hyperlipidemia (Mogensen et al., 2017), history of hormone medication (Li et al., 2018b), decompression sickness (Jones et al., 1993), and blood system diseases such as Gaucher disease (Reed et al., 2018) and sickle cell anemia (Severyns and Gayet, 2021) among others. The loss of blood supply to bone tissue leads to a decrease in the activity of bone cells, which leads to bone destruction (Zhou et al., 2019).

The destruction of bone activates the self-repair response of bone tissue (Shrivats et al., 2014) [including vascular regeneration, new bone formation, and sequestered bone resorption (Mont et al., 1998)]. However, the self-repair response of bone tissue is hindered by the harsh microenvironment of the osteonecrosis site (Zheng et al., 2015). Ineffective repairs such as fibrous tissue repair cannot replace the original bone tissue in terms of structure and support performance. Therefore, osteonecrosis involving joints often leads to the gradual collapse of the articular surface, which in turn causes arthritis (Calder et al., 2001; Hernigou et al., 2020; Cui et al., 2021).

At present, in both basic research on and clinical application of osteonecrosis treatments, there are a large number of studies that report various constructive methods. Systemic management may be unsuitable for osteonecrosis because the unavoidable problems of systemic treatment include insufficient local osteonecrosis concentration and systemic side effects. Recently, researchers to treat osteonecrosis locally through injection of stem cells (Piuzzi et al., 2017), growth factors (Rackwitz et al., 2012; Peng and Wang, 2017), cytokines (Rackwitz et al., 2012; Peng and Wang, 2017; Yang et al., 2018), various drugs (Feng et al., 2017; Guo et al., 2017; Huang et al., 2018), and hormones (Bakhshi et al., 2012; Zhou et al., 2017) at the site of osteonecrosis to achieve local treatment of osteonecrosis. These basic researchs have had varying outcomes. However, such local injection therapies face problems such as leakage, burst release, and loss of biological activity (Li et al., 2009; Tai et al., 2013; Phipps et al., 2016).

In clinical application, surgical treatment is the most important means. Surgical therapies, including core decompression, osteotomy, arthroplasty, and so forth, have been developed in clinical practice, but they are also subject to limitations, such as the narrow scope of application and repeated revision operations (Cao et al., 2016). At present, for the early stage of osteonecrosis, core decompression combined with bone grafting is widely practiced (Andronic et al., 2021). Both autologous bone grafting and allogeneic bone grafting meet the surgical needs. However, autologous bone grafting is limited by insufficient donor supply and secondary damage to and complications at the donor site, and allogeneic bone grafting faces issues such as immune rejection. These problems limit the clinical application of bone grafting (Lord et al., 1988; Stevenson et al., 1996).

Thanks to the development of biotechnology and materials science, many biomaterials are available to make bone substitute materials to cope with the problems in bone grafting. Among them, polymers are widely used in the study of osteonecrosis because of their excellent biocompatibility and biodegradability. This article focuses on applying various polymers in osteonecrosis and elaborates and summarizes the current research. Then, the different functions of various polymers combined with different substances are discussed. Finally, the application of polymers in the treatment of osteonecrosis and future outlook are summarized. We aim to provide a comprehensive review of the application of polymers in the treatment of osteonecrosis and a meaningful theoretical basis further to advance the treatment of osteonecrosis with biomedical polymer materials.

## Polymers Used in Osteonecrosis Therapy

Biocompatibility, biodegradability, and certain mechanical properties are required for biomaterials used in osteonecrosis research (Zhu et al., 2020). Biocompatibility is the primary criterion for tissue engineering materials. It allows cell adhesion, migration, and proliferation without triggering an immune response and severe inflammation (Williams, 2008). Appropriate biodegradability and certain mechanical properties are also characteristics that osteonecrosis repair biomaterials need to have. Biomaterials with appropriate biodegradability and mechanical properties provide sufficient support before new bone is formed in the osteonecrosis area to avoid articular surface collapse and pathologic fracture. Biomaterials are degraded to a certain extent over time, providing enough space to form new bone tissue (Zhang et al., 2019a; Zhu et al., 2020). In addition, this feature also allows biomaterials to be used as carriers of small molecules (Phipps et al., 2016; Zhang et al., 2019a; Zhu et al., 2020). When biomaterials are degraded, the contained substances are released into the environment. The degradation of biomaterials and the absence of any toxic byproducts also avoid the body’s immune response to foreign substances and subsequent inflammation (Zhang et al., 2019a; Zhu et al., 2020).

Polymers are mainly divided into two types, natural and synthetic (Zhang et al., 2019a; Zhang et al., 2019b). At present, natural polymers such as alginate, chitosan, and peptide chain hydrogels have been used in various forms for research on osteonecrosis. Because of their inherent extracellular matrix structure, these natural polymers exhibit better biological properties than synthetic polymers in terms of cell proliferation and differentiation and hydrophobicity (Chen et al., 2011). However, synthetic polymers also have some advantages, including better mechanical strength, higher processing capability, and a more controllable degradation rate than natural polymers.

Polymers that are often used in the treatment of osteonecrosis are poly (lactide-co-glycolide) (PLGA), poly (ε-caprolactone) (PCL), polylactide (PLA), poly (propylene fumarate) (PPF) (Chen et al., 2011; Zhu et al., 2020). Various materials have different advantages and disadvantages. Furthermore, it is difficult for a single polymer to meet the requirements of suitable biocompatibility, biodegradability, porosity, and certain mechanical support properties at the same time. In order to overcome these limitations, natural polymers, synthetic polymers, cells, small molecule drugs, and other substances are combined (Table 1). These hybrid biomaterials combine the advantages of various materials to meet more requirements, such as better biological activity, more robust mechanical properties, more controllable degradation, and more convenient manufacturing capability (Lee et al., 2014b; Feng et al., 2019).

**TABLE 1:** Combined application of polymers.

Polymer	Additional material combined with polymers	Biologically active factor	Properties	References
Chitosan	Alginate	BMMSC and EPCs	Biocompatibility, porosity, low cytotoxicity and excellent cell adhesion, enhanced bone production and angiogenesis, reduced fat production	Xu et al. (2021)
Alginate (ALG)	—	SMSCs	Biocompatibility, biodegradability, osteogenesis, injectability, elasticity	Chen et al. (2014)
Cervi cornus colla (CCC)	Deproteinized bone	—	Biocompatibility, diameter 15 mm, thickness 3.5 mm, cylindrical shape, pore structure, porosity (72.86 ± 5.45%), compressive strength 4.45 ± 1.02 MPa, degradation rate after 6 weeks is 35.81%, osteogenesis	Wang et al. (2019b)
HA	CAP	—	Biocompatibility, biodegradability, osteoconductivity, bone conductivity, promotes osteogenic differentiation and bone regeneration	Wang et al. (2018)
Peptide-based hydrogel	—	BMP-2	Biocompatibility, biodegradability, glue is also liquid in different environments, osteogenesis	Phipps et al. (2016)
DBM	—	BFGF, BMP-2	Biocompatibility, enhanced osteogenesis and angiogenesis, cell adhesion	Peng and Wang, (2017)
PLGA	β-TCP	5% Mg	Biocompatibility, biodegradability, pore size PT 423.1 ± 77.0, PT 5 M 418.7 ± 33.4, PT, 10M 392.5 ± 30.2, PT 15M 411.5 ± 26.9, porosity PT 59.1 ± 9.7, PT 5M 59.4 ± 3.1, PT 10M 62.4 ± 5.3, PT 15M 65.8 ± 8.0, the connectivity is 100%, compressive strength, PT 1.5 ± 0.1 MPa, PT 5M 2.9 ± 0.2 MPa, PT 10M 3.1 ± 0.2 MPa, PT 15M 3.7 ± 0.2 MPa, osteogenesis	Lai et al. (2019)
		10% Mg		
		15% Mg		
PLGA	TCP	Icaritin	Biocompatibility, biodegradability, pore structure, compressive strength 47.03 ± 33.58 N, enhanced bone formation	Qin et al. (2015)
PLGA	CPC	BMP, VEGF	Biocompatibility, biodegradability, porosity 62.13 ± 4.28%, compressive strength of 6.60 ± 1.02 MPa, osteogenic, angiogenic	Zhang et al. (2016)
PCL	TCP	BMMCs	Biocompatibility, pore structure, porosity near section is 15%, the middle section is 40%, the far section is 16%, the 8-weeks degradation rate of the proximal segment 42.5 ± 14.0%, 5.3 ± 1.9% at the middle segment, 5.5 ± 3.2% at the distal segment, osteogenic, vascular	Maruyama et al. (2018)
PLA	Nano-hydroxyapatite, collagen	BMMSC	Biocompatibility, biodegradability, pore size of 300 ± 250 µm, porosity of 70–90%, vascularity, osteogenesis	Wang et al. (2019a)
PPF	CPC	Ginsenoside Rg1	Biocompatibility, biodegradability, pore structure, the compressive strength in C/P = 0, C/P = 1 and C/P = 2 groups are 13.66 3.00 MPa, 15.68 3.52 MPa and 21.37 1.06 MPa, respectively, osteogenic, angiogenic	Chang et al. (2010)

BMMSC, Bone marrow mesenchymal stem cells; EPCs, endothelial progenitor cells; SMSCs, synovial fluid mesenchymal stem cells; HA, hyaluronic acid; CAP, calcium phosphate; BMP-2, bone morphogenetic protein-2; DBM, demineralized bone matrix; BFGF-2, basic fibroblast growth factor-2; β-TCP, β-tricalcium phosphate; Mg, magnesium; CPC, calcium phosphate; VEGF, vascular endothelial growth factor.

### Natural Polymers in Osteonecrosis Therapy

Because natural polymers have the characteristics of biocompatibility and biodegradability—and the biological functional molecules on the surface of natural polymers are conducive to cell adhesion, aggregation, proliferation, and differentiation—natural polymers are widely used in tissue engineering. Various natural polymers such as chitosan, alginate, and peptide chain hydrogel have been made into bone substitute materials for osteonecrosis therapy (Zhang et al., 2019a).

#### Chitosan and Its Derivatives

**FIGURE 1:**
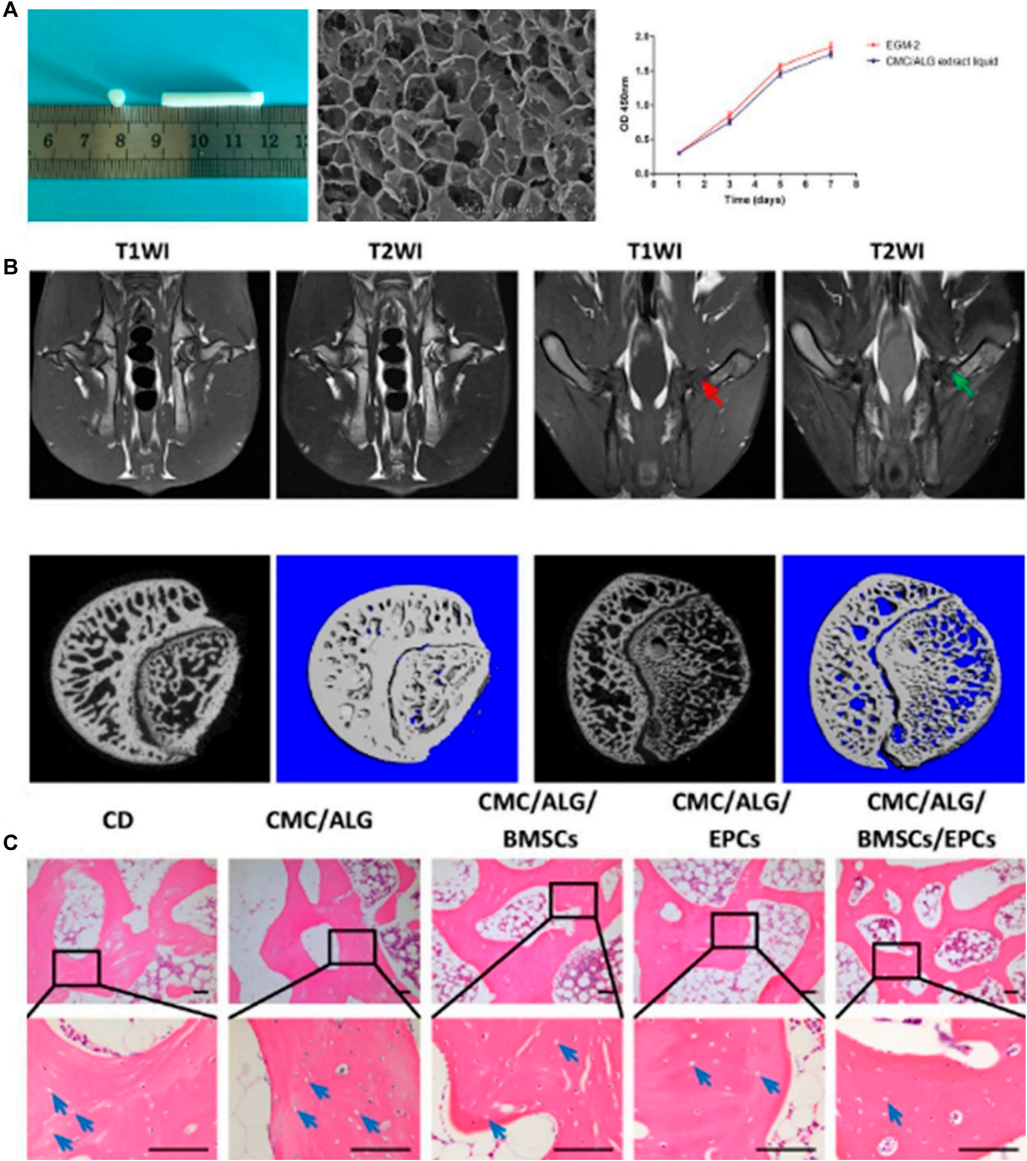
BMMSC and EPCs dual-loaded CMC/ALG scaffolds for enhanced bone regeneration in ONFH (Xu et al., 2021). **(A)** Morphology of the scaffold for *in vivo* transplantation. The SEM micrograph showed the porous structure of the scaffold. And CCK-8 assay results confirmed that CMC/ALG scaffolds generated no cytotoxicity effects on BMSC/EPC viability. **(B)** Representative MRI photograph of the normal rabbit and the rabbit model. 2D and 3D micro-CT images of the femoral head in normal group and model group. **(C)** H&E staining of the empty lacuna in the necrotic region of the femoral head for each group. Reproduced with permission (Xu et al., 2021). Copyright 2021, Wiley Periodicals LLC.

Chitosan is a linear polycationic polysaccharide polymer derivative of chitin. A large number of research reports show that chitosan has good biocompatibility, high biodegradability, low allergenicity, antibacterial properties, and wound healing activity. Chitosan can be easily extracted from shellfish and other seafood waste. However, the poor solubility of chitosan in neutral and alkaline media limits the direct application of chitosan in medicine and biomedicine (Prabaharan and Mano, 2005; Bhattarai et al., 2010; Fonseca-Santos and Chorilli, 2017). Chitosan is easily carboxy-methylated to produce carboxymethyl chitosan (CMC). The solubility of CMC in aqueous media is greatly enhanced, while biodegradability and biocompatibility are maintained. Moreover, CMC is easy to modify chemically and has a high affinity for macromolecules in the body. CMC is widely used in biomedicine and various tissue engineering fields (Fonseca-Santos and Chorilli, 2017). Xu et al. (2021) manufactured a carboxymethyl chitosan/alginate scaffold (CMC/ALG) by a lyophilization approach and loaded BMMSC and EPCs on the scaffold. Their research confirmed that the scaffold has good biocompatibility (Figure 1A) (porosity, low cytotoxicity, and excellent cell adhesion). The researchers established a rabbit model of steroid-induced osteonecrosis of the femoral head (ONFH) (Figure 1B). After CD was performed on the necrotic femoral head, a CMC/ALG/BMMSC/EPC scaffold was implanted into the rabbit femoral head. Two weeks later, the results of radiological evaluation and histological analysis showed that the CMC/ALG/BMMSC/EPC group achieved the best curative effect in the repair of osteonecrosis in each group (Figure 1C). They observed that the CMC/ALG/BMMSC/EPC group had significant bone formation and angiogenesis and decreased fat production, which promoted the repair of ONFH.

#### Alginate

Alginate is a frequently used biomedical material, often for drug delivery, cell embedding, tissue embedding, and cartilage tissue regeneration (Chen et al., 2014). Alginate has good water solubility and good biocompatibility and can be made into a gel (Majima et al., 2005). Alginate is often made into an injectable gel for surgery (Goodship and Birch, 2005), and many researchers use it for research on osteonecrosis repair.

**FIGURE 2:**
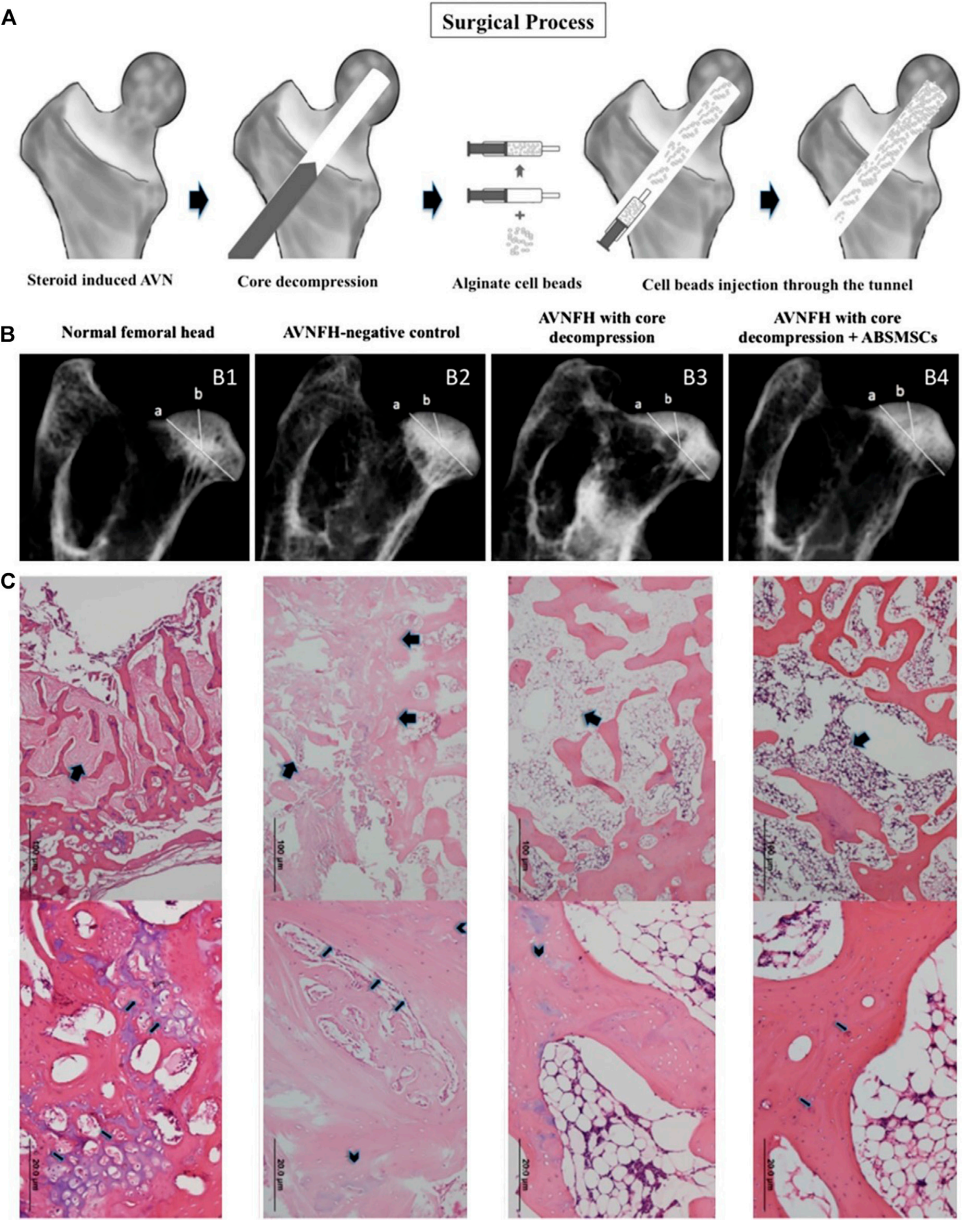
The alginate beads loaded with SMSCs promote bone formation and angiogenesis in animal models of femoral head necrosis (Chen et al., 2014). **(A)** Procedures for treating femoral head necrosis in the animal model. Radiographs of harvested femoral heads **(B)** and histological observations **(C)** of different groups. Reproduced with permission (Chen et al., 2014). Copyright 2014, ELSEVIER.

Chen et al. (2014) embedded synovial fluid mesenchymal stem cells (SMSCs) in alginate beads and observed the biological activity and osteogenic differentiation of synovial mesenchymal stem cells in the internal environment of alginate beads in *in vitro* experiments. The alginate beads embedded with synovial fluid MSCs (ABSMSCs) were implanted into the femoral head of the rabbit model of hormone-induced femoral head necrosis after core decompression surgery (Figure 2A).

The results of *in vitro* experiments show that the SMSC in the internal environment of alginate beads have the potential to differentiate into bone. *In vivo*, the hormone-induced femoral head necrosis rabbit model can be treated by core decompression and alginate beads carrying ABSMSCs implantation. This method maintains the density and spherical shape of the femoral head and promotes bone regeneration within the necrotic femoral head (Figure 2B). Histological analysis results also showed that compared with other groups, the ABSMSCs group had more new bone tissue and new blood vessels in the area of osteonecrosis (Figure 2C). Therefore, alginate is also an polymer suitable for osteonecrosis research.

#### Cervi Cornus Colla

**FIGURE 3:**
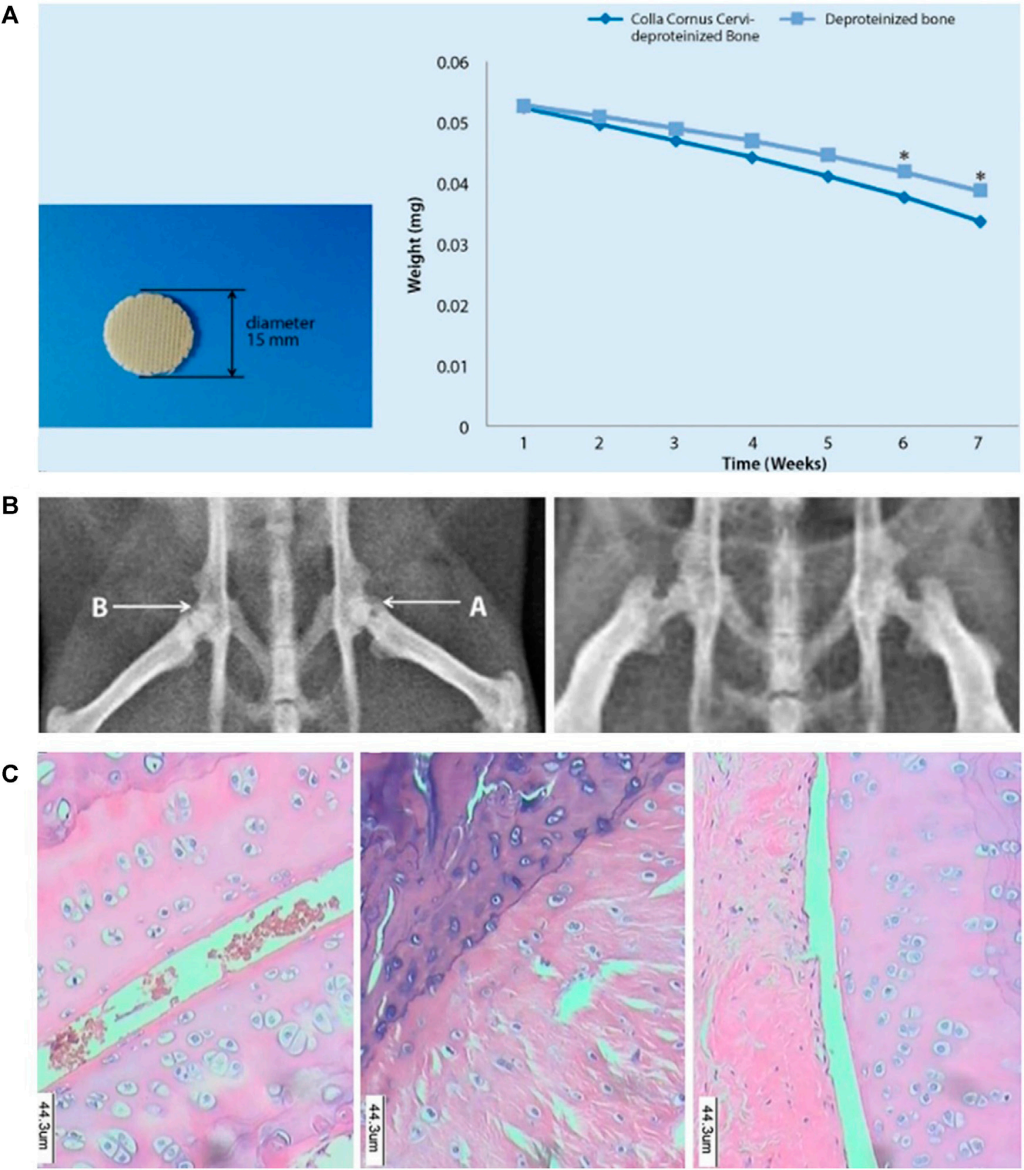
CCC deproteinized bone scaffold reduced femoral head necrosis in rats. **(A)** Exterior view of a 3D printed CCC-deproteinized bone scaffold, and scaffold degradation curves. The degradation levels of the CCC-deproteinized bone scaffolds and deproteinizedbone scaffolds after 6 weeks immersion in PBS reached 35.81 and 26.61%, respectively (*p < 0.05). **(B)** The femoral head implanted with a CCC-deproteinized bone scaffold and the non-implanted femoral head exhibited different outcome. **(C)** Pathological observation of the femoral head of rat in different groups. Significant alleviation of femoral head necrosis was observed in the rats implanted with CCC-deproteinized bone scaffolds. Reproduced with permission (Wang et al., 2019b). Copyright 2019, SPRINGER.

Cervi Cornus colla (CCC) is a Chinese medicine extracted from deer antlers, and it is a protein-polysaccharide complex. CCC contains 16 amino acids, including glycine, proline, glutamic acid, and so forth. (Choi et al., 2013). CCC has long been used in animal model tests and clinical human experiments. CCC has been used to prevent and treat acute and chronic arthritis, osteoporosis, fractures, hypercholesterolemia, and other diseases (Kim et al., 2013; Li et al., 2014). Wang et al. (2019b) processed the proximal pig femur to obtain a deproteinized bone meal. After mixing the deproteinized bone meal with CCC and synthetic organic materials, CCC-deproteinized bone scaffolds were made by 3D printing technology. The 3D printed CCC-deproteinized bone scaffold had a porous structure, degradability, and excellent mechanical properties (Figure 3A). They implanted the scaffold into the femoral head of the mouse model with osteonecrosis of the femoral head (ONFH) to repair osteonecrosis (Figure 3B) and found that the CCC deproteinized bone scaffold significantly reduced femoral head necrosis in rats (Figure 3C). *In vitro* experiments also showed that osteoblasts aggregated and adhered in the pore structure of the CCC deproteinized bone scaffold, and the CCC-deproteinized bone scaffold enhances the proliferation of osteoblasts (Wang et al., 2019b).

#### Hyaluronic Acid

**FIGURE 4:**
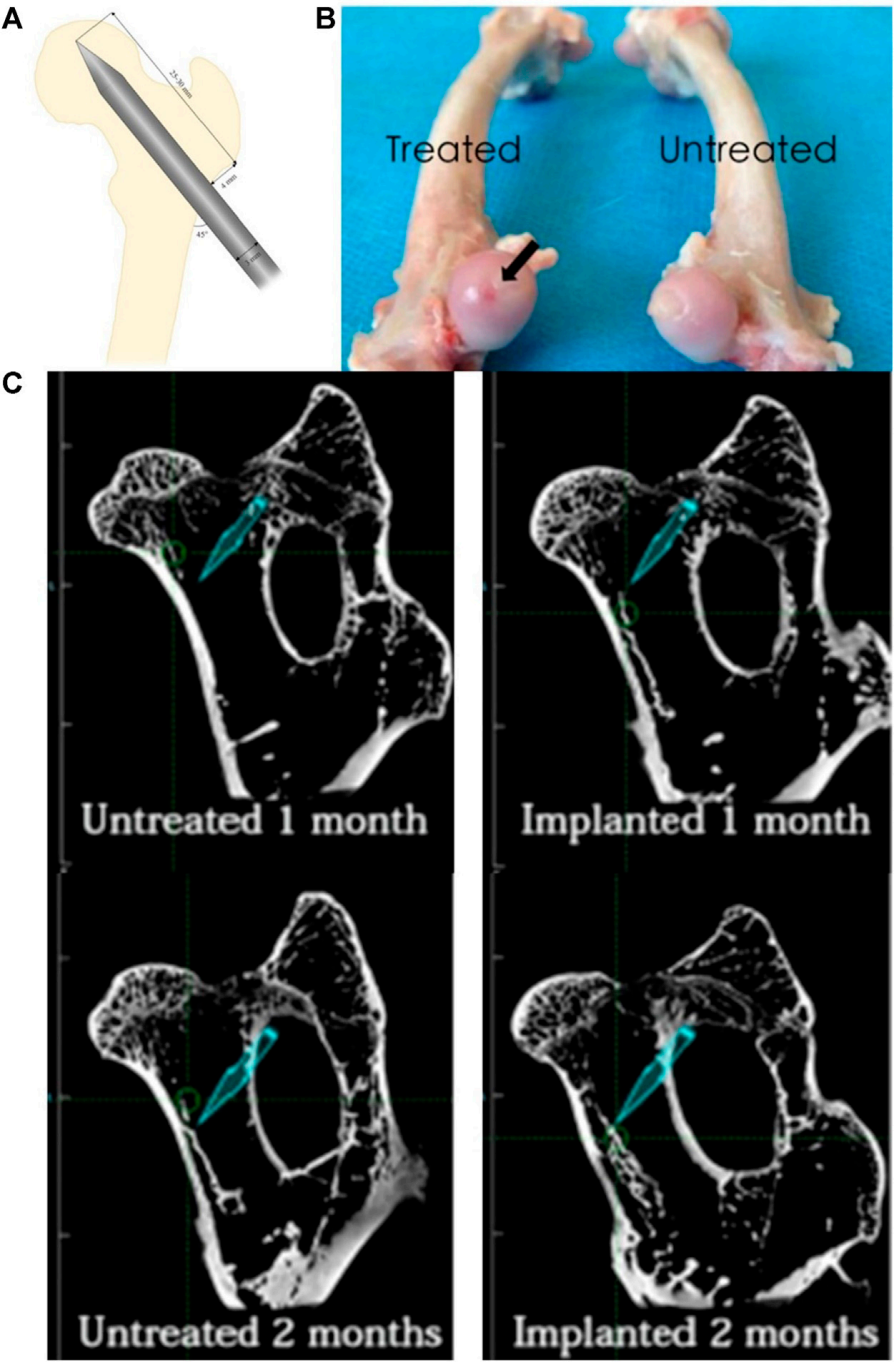
HA-BP/CAP composite hydrogel can promote bone regeneration at the site of osteonecrosis (Wang et al., 2018). **(A)** The details regarding the standard animal model of ONFH. **(B)** The subchondral bone of the femoral head in the experimental group presented the establishment of the ONFH animal model because of the dark red area on the surface of the femoral head, while the untreated group appeared normal. **(C)** shows that the amount of bone regeneration at 1 and 2 months after injection of HA-BP/CAP composite hydrogel in the experimental group was significantly greater than that in the control group. Reproduced with permission (Wang et al., 2018). Copyright 2018, 2018 Elsevier Inc.

Hyaluronic acid (HA), a natural polysaccharide composed of D-glucuronic acid and D-N-acetyl glucosamine repeating units (Kutlusoy et al., 2017; Rezaeeyazdi et al., 2018), is the main component of the extracellular matrix and an essential structural element in various tissues. HA plays an essential role in angiogenesis and wound healing (Wu et al., 2017; Rezaeeyazdi et al., 2018), and researchers are currently using HA to study osteonecrosis. Wang et al. (2018) combined bisphosphonate (BP)-modified HA (HA-BP) and CAP to create an HA-BP/CAP composite hydrogel. Their *in vitro* experiments confirmed that the composite hydrogel has good biocompatibility. The composite hydrogel material was also injected into the femoral skull tunnel of the ONFH rabbit model as the experimental group. The control group was injected with saline (Figures 4A,B). At 1 month and 2 months, the repair of femoral head necrosis of the two rabbit models was compared. As expected, in the radiological evaluation and histological analysis, the experimental group produced more new bone mineral tissue than the control group (Figure 4C), demonstrating that this HA-BP/CAP composite hydrogel can promote bone regeneration at the site of osteonecrosis.

#### Peptide-Based Hydrogels

**FIGURE 5:**
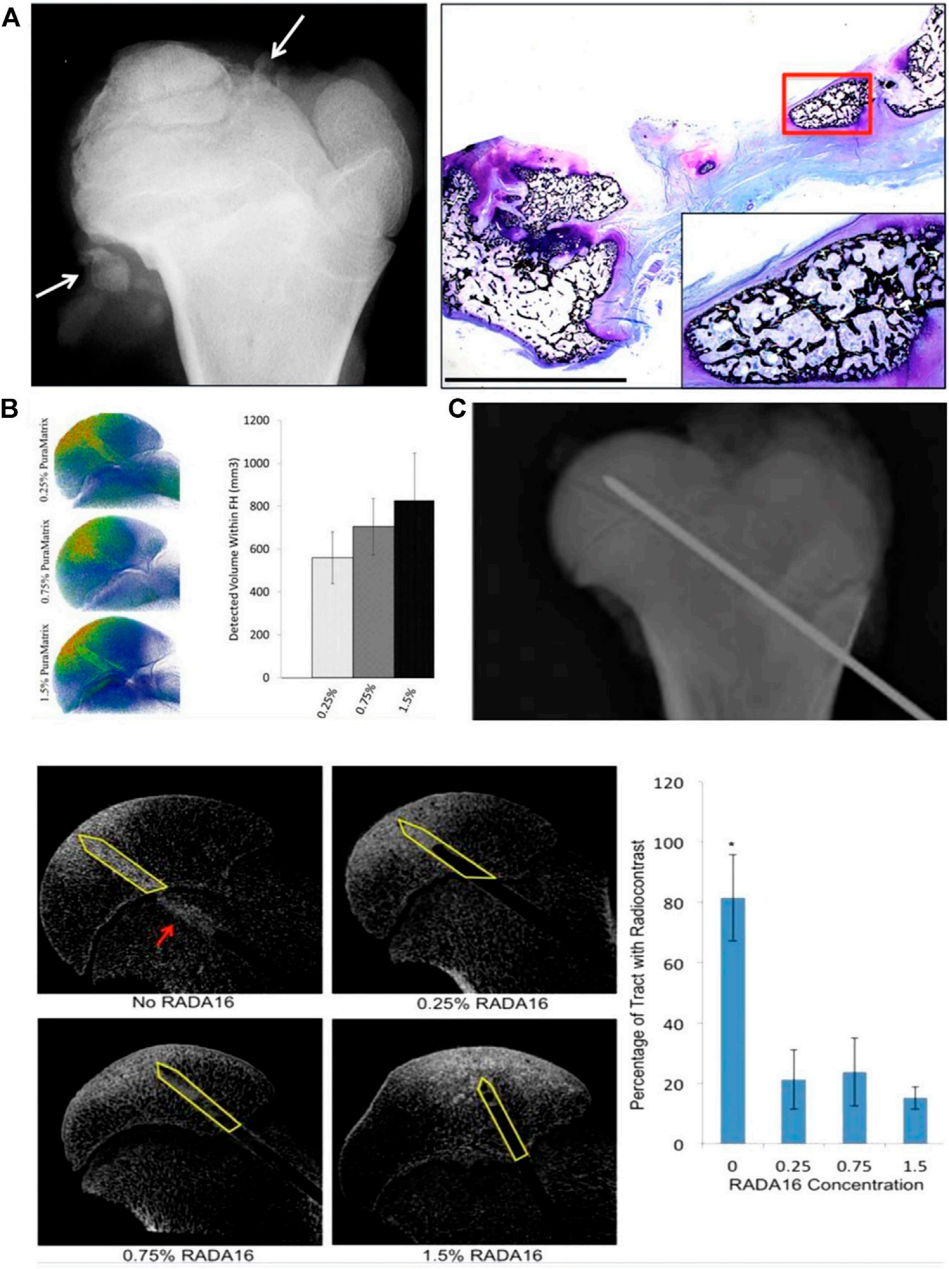
A peptide-based hydrogel named RADA16 provides a feasible method for the leakage problem encountered by local injection of BMP-2 in the treatment of femoral head necrosis (Vandermeer et al., 2011; Phipps et al., 2016). **(A)** Radiograph and histological analysis showed heterotopic ossification after local intraosseous administration of ibandronate and BMP-2 (Vandermeer et al., 2011). **(B)** Distribution of different concentrations of RADA16/radiocontrast mixture in the femoral head (Phipps et al., 2016). **(C)** Intraosseous needle introduced in the central region of the femoral head by transphyseal approach for RADA16/radiocontrast infusion (Phipps et al., 2016). **(D)** Micro-CT images showing different amount of backflow of radiocontrast solution down the needle track. Bar graph showing percentage of needle track with radiocontrast backflow after the removal of the needle (Phipps et al., 2016). Reproduced with permission (Vandermeer et al., 2011). Copyright 2011, LIPPINCOTT WILLIAMS & WILKINS. Reproduced with permission (Phipps et al., 2016). Copyright 2016, American Chemical Society.

Some peptide and protein nanofiber structures have also been extensively studied as biomaterials. As early as 2011, Vandermeer et al. (2011) injected ibandronate combined with BMP-2 into an animal model of ischemic femoral head necrosis. Experiments confirmed that ibandronate combined with BMP-2 could reduce femoral head deformities and at the same time stimulate bone formation. However, they also found that infusion of BMP-2 solution can cause the unnecessary spread of BMP-2 outside the femoral head and produce heterotopic ossification in the hip joint capsule (Figure 5A).

Five years later, Phipps et al. (2016), in the same laboratory as Vandermeer *et al.*, used a peptide-based hydrogel called RADA16 to provide a solution to the BMP-2 leakage problem previously encountered by. They believe that this novel method may provide benefits for osteonecrosis therapy.

RADA16 is a peptide-based hydrogel composed of 16 amino acids (Yokoi et al., 2005) and has a β-sheet structure in a saline environment (Zhang et al., 1993). Previous studies have shown that this peptide-based hydrogel is biocompatible, biodegradable, and can support new bone formation (Misawa et al., 2006; Nakahara et al., 2010; Kohgo et al., 2011). In their *in vivo* and *in vitro* experiments, Phipps et al. (2016) used RADA16 as a carrier to deliver BMP-2, retaining the biological activity of BMP-2 and effectively controlling the diffusion of BMP-2 (Figures 5B,C). After a mixed injection of RADA16 and a radiographic agent, the backflow of the contrast agent in the porcine femoral head channel was significantly reduced (Figure 5D).

The above findings support peptide-based hydrogel as an intraosseous carrier and provide a new solution to the leakage problem in osteonecrosis therapy. They also guide the next steps in studying peptide chain hydrogel in osteonecrosis model experiments.

#### Demineralized Bone Matrix

The bone tissue removes the mineralized components and retains the organic matrix and growth factors to obtain demineralized DBM. DBM has strong osteogenic properties because it contains many organic components and growth factors and is often used in bone repair research (Lee et al., 2014a). BFGF can affect gene expression and angiogenesis. Therefore, it is considered to be a critical factor in the process of bone repair (Hu et al., 2015). Peng and Wang (2017) transfected adenovirus-mediated bone morphogenetic protein 2 (Ad-BMP-2) and bFGF into BMMSC. The modified bone marrow mesenchymal stem cells combined with DBM (Ad-BMP2-bFGF-GFP group) were then implanted into an ONFH canine model. This experiment shows that the BMMSC modified by Ad-BMP-2/bFGF combined with DBM can repair the osteonecrosis of the femoral head in the ONFH canine model by promoting bone formation and angiogenesis, and DBM itself has osteoinduction and osteoconduction capabilities. Both radiological evaluation and histological analysis show that the Ad-BMP2-bFGF-GFP group had a larger area of new bone and a higher density of new blood vessels than the other groups.

### Synthetic Polymers in Osteonecrosis Therapy

In recent years, synthetic polymers have received more attention than natural polymers because of their desirable properties in bone engineering, such as porosity, degradation time, and mechanical properties. They have strong shaping abilities and can be made into various shapes according to need (Oh, 2003; Sheikh et al., 2016).

#### Poly (Lactide-Co-Glycolide)

**FIGURE 6:**
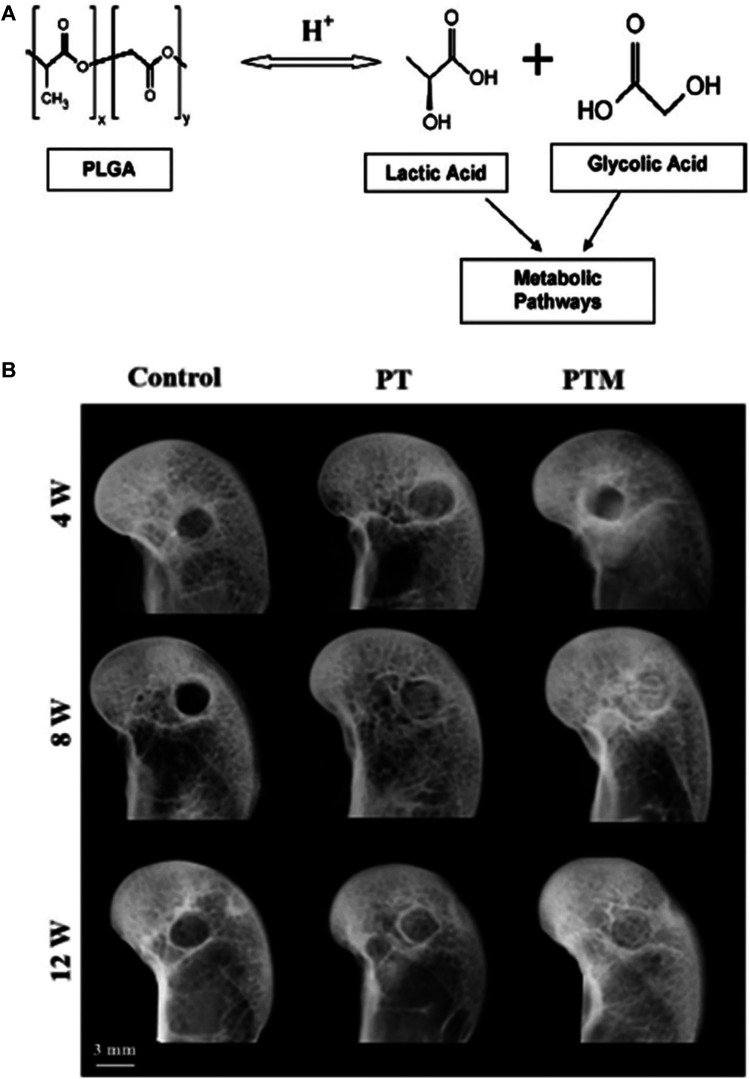
PLGA for osteonecrosis therapy. **(A)** Hydrolysis of PLGA (Danhier et al., 2012). **(B)** Representative radiographs showed that new bone formation within bone tunnel at 4, 8, and 12 weeks after surgery (Lai et al., 2019). Reproduced with permission (Danhier et al., 2012). Copyright 2012, Elsevier Ltd. Reproduced with permission (Lai et al., 2019). Copyright 2019, Elsevier Ltd.

Poly (lactide-*co*-glycolide) (PLGA) is currently one of the most successfully developed synthetic biodegradable polymers. Because of its excellent biocompatibility and biodegradability, it has been widely used in research on various human delivery systems (Danhier et al., 2012). PLGA is approved by the U.S. Food and Drug Administration (FDA) and the European Medicines Agency for use in various human drug delivery systems (Kempen et al., 2008). PLGA has extremely low toxicity in the human body because the hydrolyzed metabolites of PLGA are monomeric lactic acid and monomeric glycolic acid, as shown in Figure 6A. As endogenous substances in humans, these two monomers are easily metabolized through the human body’s Krebs cycle (Kumari et al., 2010).

Lai et al. (2018) established a poly (lactide-coglycolide) (PLGA), β-TCP composite scaffold using low-temperature rapid prototyping (LT-RP) technology. The PLGA/TCP (PT) scaffold has a trabecular pore structure with good biocompatibility, bone conductivity, and biodegradability *in vivo* and *in vitro*. Lai et al. (2019) also added magnesium (Mg) to the PT scaffold to make a PTM scaffold in a follow-up study. The research results show that the PTM scaffold has a good bionic structure and suitable mechanical properties. The PTM scaffold has the dual capabilities of osteogenesis and angiogenesis (Figure 6B). The PTM scaffold is synergistic in enhancing the formation and quality of new bone in the rabbit model of steroid-associated osteonecrosis (SAON), and it has a stronger ability to promote bone formation than the PT scaffold.

Qin et al. (2015) added icaritin to the PLGA/TCP scaffolds to produce PLGA/TCP/icaritin (PTI) scaffolds. A steroid-associated osteonecrosis (SAON) animal model was established, and the PTI and PT scaffolds were implanted in the animal model. A non-implanted scaffold group was the control group. The effects of the PTI scaffold on the recruitment, bone formation, and anti-adipogenesis of bone marrow mesenchymal stem cells (BMMSC) were observed. The results of the study showed that the incidence of femoral head collapse in the PTI stent group was the lowest. Compared with the control group and the PT group, the femoral head cartilage was better preserved in the PTI scaffold group, and more new bone was formed in the bone tunnel.

Zhang et al. (2016) produced a composite PLGA microsphere. This calcium phosphate (CPC) scaffold contained BMP-vascular endothelial growth factor (VEGF)-loaded PLGA microspheres (BMP-VEGF-PLGA-CPC) and exhibited compressive strength equivalent to that of cancellous bone. The composite microspheres showed good biocompatibility and promoted bone formation and angiogenesis in animal experiments. Compared with other scaffold groups, more new mineralized tissue can be observed around the scaffold, and more new blood vessels appear in the newly mineralized tissue in the BMP-VEGF-PLGA-CPC group. Zhang et al. (2016) proposed that the BMP-VEGF-PLGA-CPC scaffold has a potentially useful application in the treatment of osteonecrosis.

In summary, PLGA is currently one of the most widely used synthetic polymer materials in the field of osteonecrosis research. We believe that PLGA will continue to receive more attention in this field in the future.

#### Poly (ε-Caprolactone)

Poly (ε-caprolactone) (PCL) is another polymer material that is widely used in bioengineering. The biocompatibility of PCL is excellent, and the surface chemistry of PCL is suitable for cell attachment, proliferation, and differentiation. Moreover, the degradation byproducts of PCL are non-toxic and can usually be metabolized and eliminated through the body’s natural metabolic pathways (Gao et al., 2017). A previous study (Sung et al., 2004) compared the pH level of the environment around the implant after PCL and PLGA scaffolds were implanted under the skin of the mouse back. The study concluded that PCL is less likely to acidify the environment than PLGA and less likely to cause inflammation in the body. Thermoplastic polymers such as PCL can be easily produced by 3D printing technology into controllable, various-shaped, porous scaffolds for various scientific research (Lam et al., 2009; Wang et al., 2019c). However, the degradation rate of PCL in the abdomen of rats is low, and the low degradation rate causes PCL to hinder the production of new cell tissue in the implantation area and may even trigger the body’s immune rejection reaction (Qazi et al., 2014; Kargozar et al., 2018; Zhu et al., 2020).

**FIGURE 7:**
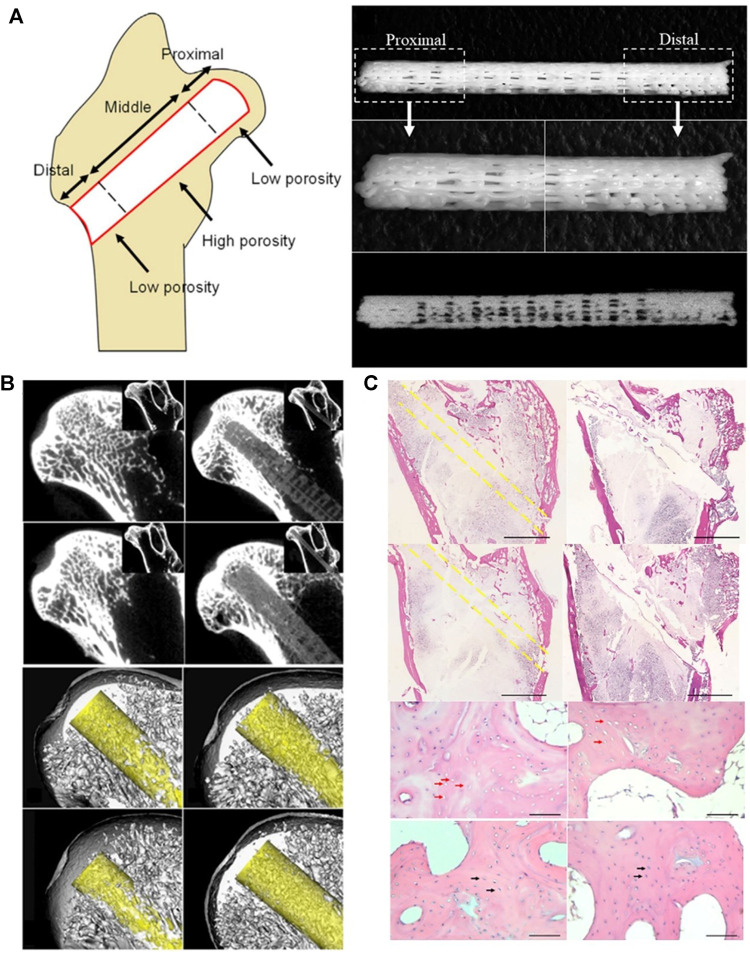
FGS and FGS/BMMC could promote bone regeneration in the area of osteonecrosis. **(A)** A schematic image shows how the three segments of FGS with different porosities are distributed in the femoral head. The FGS consisting of three segments of spatially graded porosity, including 4 mm length proximal segment of 15% porosity, 17 mm length middle segment of 40% porosity, and 6 mm distal segment of 15% porosity. **(B)** FGS is degraded at the proximal end, and there is mineralized tissue around it. **(C)** Histological analysis confirmed that there was more new bone formation around FGS than other groups. Reproduced with permission (Maruyama et al., 2018). Copyright 2018, Elsevier Ltd.

Maruyama et al. (2018) produced PCL/TCP functionally graded scaffold (FGS), which was divided into three porosity-spatially-graded sections. The porosity of the proximal section was 15% to produce a length of 4 mm, the porosity of the middle section was 40% to produce a length of 17 mm, and the porosity of the distal section was 16% to produce a length of 6 mm. The porosity of each segment was similar to that of the human femur (Figure 7A). The study also added bone marrow-derived mononuclear cells (BMMCs) to FGS and implanted FGS + BMMCs and FGS into the femoral head of a rabbit steroid-induced osteonecrosis model after CD surgery. The results showed that the degradation rate of the proximal segment of FGS was higher than that of the middle and distal segments. The degradation rate of the proximal part of FGS in the FGS/BMMCs group was higher than that in the FGS group. The addition of TCP increased the degradation rate of PCL to a suitable range. The experimental results also showed that more new bone was formed in the bone tunnel in the FGS group than in the CD group, and the FGS/BMMCS group had the newest bone of each group. The results indicated that both FGS and FGS/BMMCS could promote bone regeneration in the area of osteonecrosis (Figures 7B,C).

#### Poly(Lactic Acid)

**FIGURE 8:**
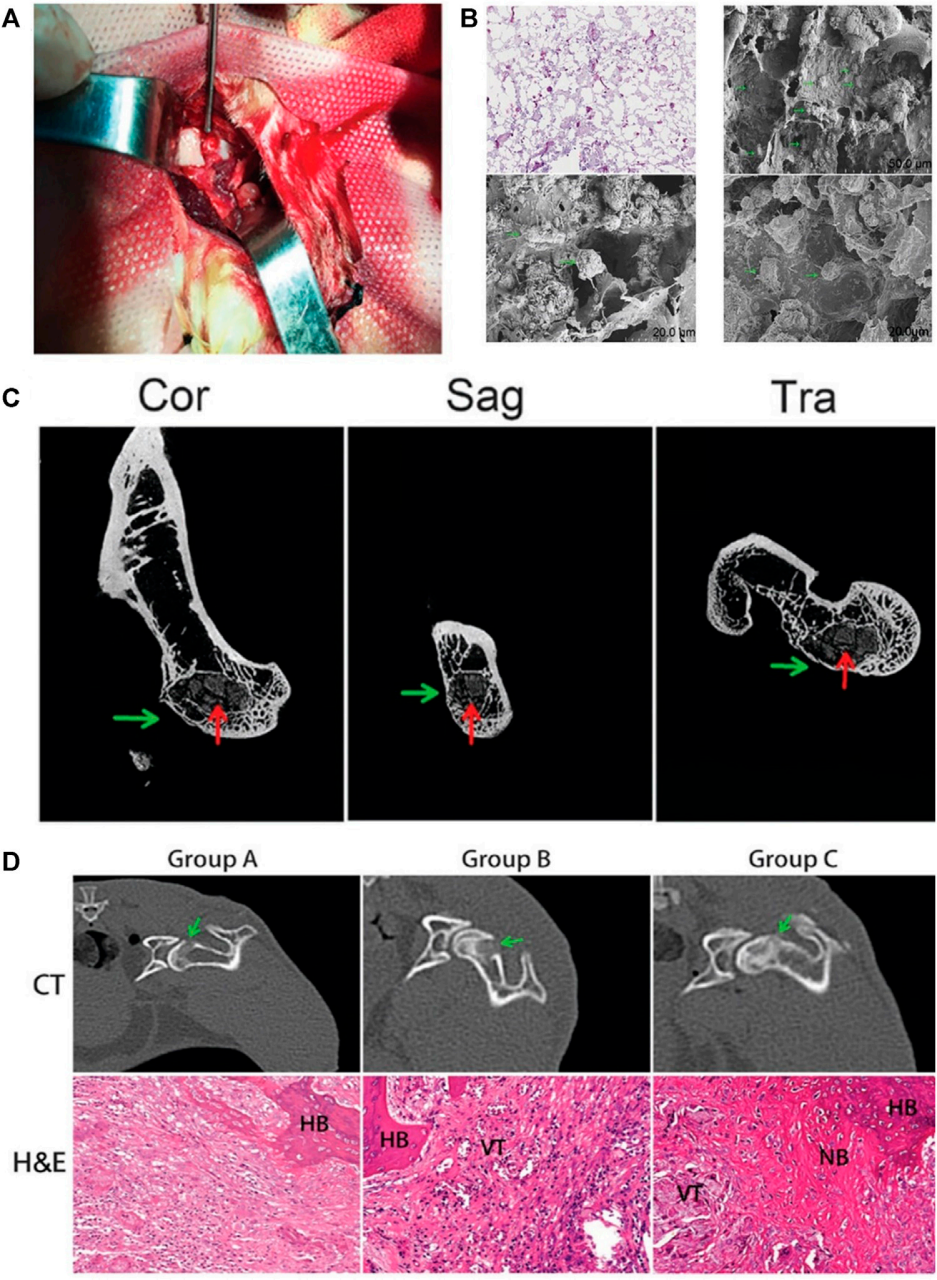
PLA-based composite scaffolds may improve the curative effect of CD and provide a strategy for treating ANFH. **(A)** composite scaffolds were implanted into the decompression tunnel. **(B)** A total of 24 h after seeding, hematoxylin and eosin staining and scanning electron microscopy micrographs revealed that BMMSC attach to scaffolds. **(C)** The micro-CT results further showed more bone trabeculae around the decompression tunnel in group C. **(D)** CT images and HE micrographs at 4 weeks post-operation. The CT images suggested that the osteogenesis in the decompression tunnel of group C was significantly higher than that in the other two groups. Histology micrographs of H&E staining of bone tunnels (x200) of the three groups. CT, computerized tomographic scanning; NB, new bone; HB, host bone; VT, vascular tissue; H&E, hematoxylin and eosin. Group A, pure CD; group B, CD + nHAC/PLA; and group C, CD + nHAC/PLA/BMMSC. Reproduced with permission (Wang et al., 2019a). Copyright 2019, Spandidos Publ Ltd.

Lactic acid, the precursor of poly (lactic acid) (PLA), is non-toxic to humans. PLA is also one of the most widely used synthetic polymers approved by the FDA for biomedical purposes (Abdal-hay et al., 2013; Zhao et al., 2019b; Zhao et al., 2021). Wang et al. (2019a) combined PLA, nano-hydroxyapatite, and collagen PLA to establish a nano-hydroxyapatite/collagen I/poly-L-lactic acid composite scaffold (nHAC/PLA). The BMMSC were cultured on the composite scaffold and implanted into the necrotic femoral head after CD in the ANFH rabbit model (Figure 8A). The researchers observed the adhesion of BMMCs to the nHAC/PLA scaffold through an electron microscope, which proved the good biocompatibility of the composite scaffold (Figure 8B). The experimental results showed that the nHAC/PLA/BMMSC group had the best therapeutic effect in the treatment of osteonecrosis. Micro-CT and histological analysis showed that the nHAC/PLA/BMMSC group produced more new bone tissue than the CD and nHAC/PLA groups, and the degradation rate of the composite scaffold was also the highest in these groups (Figures 8C,D).

#### Poly(Propylene Fumarate)

**FIGURE 9:**
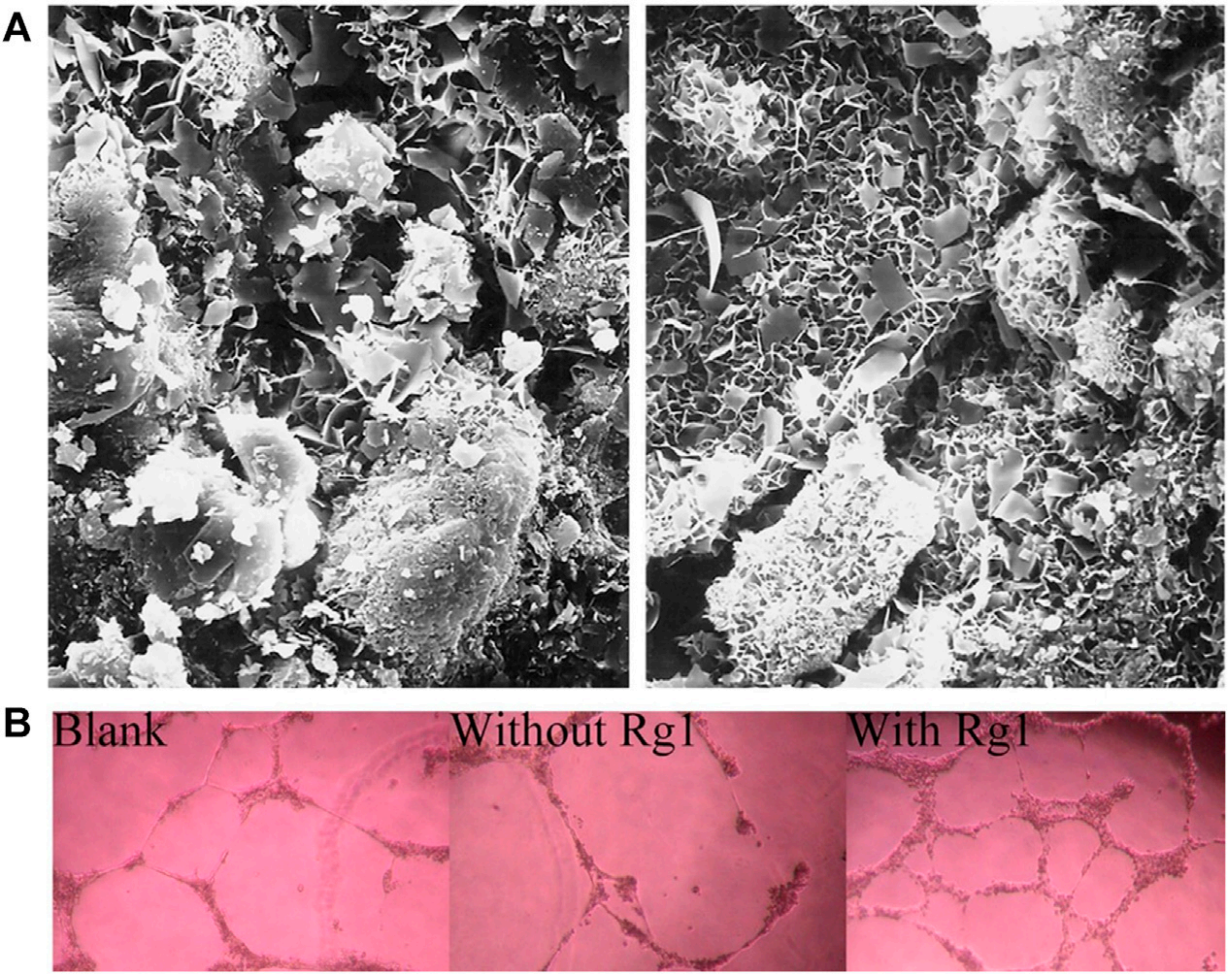
The bone cement composite material added with ginsenoside Rg1 to treat osteonecrosis. **(A)** Section morphology observation of the cement in different C/P ratio by scanning electron microscopy. **(B)** Tube formation in different extract-contained media from cements. Reproduced with permission (Chang et al., 2010). Copyright 2010, Elsevier Ltd.

In recent years, polypropylene fumarate (PPF) has attracted widespread attention as a promising biodegradable, injectable, and non-toxic bone cement material (Lee et al., 2006). Chang et al. (2010) study combined polypropylene fumarate (PPF) and CPC. They also studied the effects of different CPC/PPF ratios on their mechanical properties and cytotoxicity (Figure 9A). The results show that as the C/P ratio increases (C/P = 0, C/P = 1 and, C/P = 2), the cytotoxicity of the composite bone cement decreases, and the increase in the CPC ratio also enhances the mechanical strength of the composite bone cement. The bone cement composite material added with ginsenoside Rg1 also has an angiogenic effect (Figure 9B). They believe that this newly developed angiogenic bone cement composite material has significant development potential in treating femoral head necrosis.

## Functionalized Polymer Materials in Osteonecrosis Therapy

Osteonecrosis is a disease caused by the destruction of blood supply and decreased skeletal cell activity (Guo et al., 2014; Cui et al., 2021; Hines et al., 2021). The reconstruction of bone and blood supply in the necrotic area is the top priority in treating osteonecrosis (Zhu et al., 2020). Previously, researchers injected stem cells (Piuzzi et al., 2017), growth factors (Rackwitz et al., 2012; Peng and Wang, 2017), cytokines (Rackwitz et al., 2012; Peng and Wang, 2017; Yang et al., 2018), various drugs (Feng et al., 2017; Guo et al., 2017; Huang et al., 2018), and hormones (Bakhshi et al., 2012; Zhou et al., 2017) directly into the area of osteonecrosis to explore the role of these biological substances that have osteogenic and vascular functions in promoting the repair of osteonecrosis (Maruyama et al., 2018; Zhang et al., 2019a; Zhu et al., 2020). The researchers have achieved practical results.

However, problems related to biologically active substances such as loss of biological activity, short half-life *in vivo*, heterotopic ossification, and lack of effective support remain to be resolved (Phipps et al., 2016; Shi et al., 2017; Zhang et al., 2018a).

Various polymer scaffolds, gels, and microspheres with biocompatibility, biodegradability, porous structure, and excellent mechanical support have helped solve the above problems (Zhang et al., 2019a; Zhu et al., 2020). Adding biologically active substances such as stem cells and growth factors to pure polymers to enhance the osteogenesis and angiogenesis function of polymers is considered to be an effective strategy (Zhu et al., 2020). Polymer scaffolds can provide attachment points for biologically active substances, effective support, and slow-release capability, and these biologically active substances improve the biological activity and mechanical properties of pure polymers. The combination of different biologically active substances [such as stem cells (Fan et al., 2015; Ismail et al., 2017; Peng and Wang, 2017; Maruyama et al., 2018), growth factors (Wang et al., 2009; Garcia et al., 2012; Bai et al., 2014; Phipps et al., 2016; Zhang et al., 2016; Zhu et al., 2017; Chen et al., 2018), small molecule drugs (Tai et al., 2013; Qin et al., 2015; Salarian et al., 2017), metal ions (Salarian et al., 2017; Li et al., 2018a; Lai et al., 2019)] and polymers provides polymers with different functionalities. This strategy has also become a popular area in the research and treatment of osteonecrosis.

### Combination of Polymers and Bioactive Factors With Osteogenesis Function

The addition of bioactive factors with osteogenic function to polymers can enhance the osteogenic properties of polymers, which is conducive to the formation of new bone in the osteonecrosis area and prevents joint collapse and arthritis. Mesenchymal stem cells can differentiate into a variety of cell lines (such as bone cells, osteoblasts, and endothelial cells) (Fan et al., 2015; Hernigou et al., 2015; Sui et al., 2019). Bone marrow mesenchymal stem cells are accessible to culture and expand *in vitro* and accelerate bone regeneration by differentiating into osteoblasts (Le et al., 2020; Song et al., 2020; Zhu et al., 2020; El-Jawhari et al., 2021). Previous studies have shown that bone marrow mesenchymal stem cells secrete a variety of growth factors, cytokines, and other biologically active molecules to regulate the damage and repair process of ischemic tissue after transplantation (Herrmann et al., 2011).

In the study of PCL/TCP FGS (Figure 7A) constructed by Maruyama et al. (2018), the FGS/BMMSc group had more new bone formation than the other groups. The FGS/BMMSc group also had a higher FGS degradation rate than the FGS group. It is believed that (Maruyama et al., 2018) this result proves that BMMSc promotes osteogenesis and that proper pretreatment improves the therapeutic effect of bone marrow mesenchymal stem cells. Hypoxic preconditioning induces a compensatory response in bone marrow mesenchymal stem cells by activating endogenous mechanisms, increasing vitality, and reducing cell apoptosis during implantation (Tsai et al., 2012; Fan et al., 2015). Fan et al. (2015) implanted bone marrow mesenchymal stem cells on an absorbable collagen sponge under hypoxic conditions of 2 and 20% oxygen concentration to construct a hypoxic pretreatment functionalized absorbable sponge. They found that, compared with normoxic conditions, hypoxic pretreatment could more effectively overcome the obstacles of cell death *in vitro* and promote the survival and proliferation of bone marrow mesenchymal stem cells *in vitro*.

Some investigators studied SMSC. These cells show the ability to differentiate into bone, cartilage, and fat and can be easily obtained from the joint fluid (Morito et al., 2008). Chen et al. (2014) embedded synovial fluid mesenchymal stem cells (SMSCs) into alginate beads and implanted them into the femoral head of a rabbit model of femoral head necrosis induced by hormones (Figure 4A). Through core decompression and ABSMSCs implantation, the density and spherical shape of the femoral head of the rabbit model was maintained, and the bone regeneration within the necrotic femoral head was promoted (Figures 4B,C).

**FIGURE 10:**
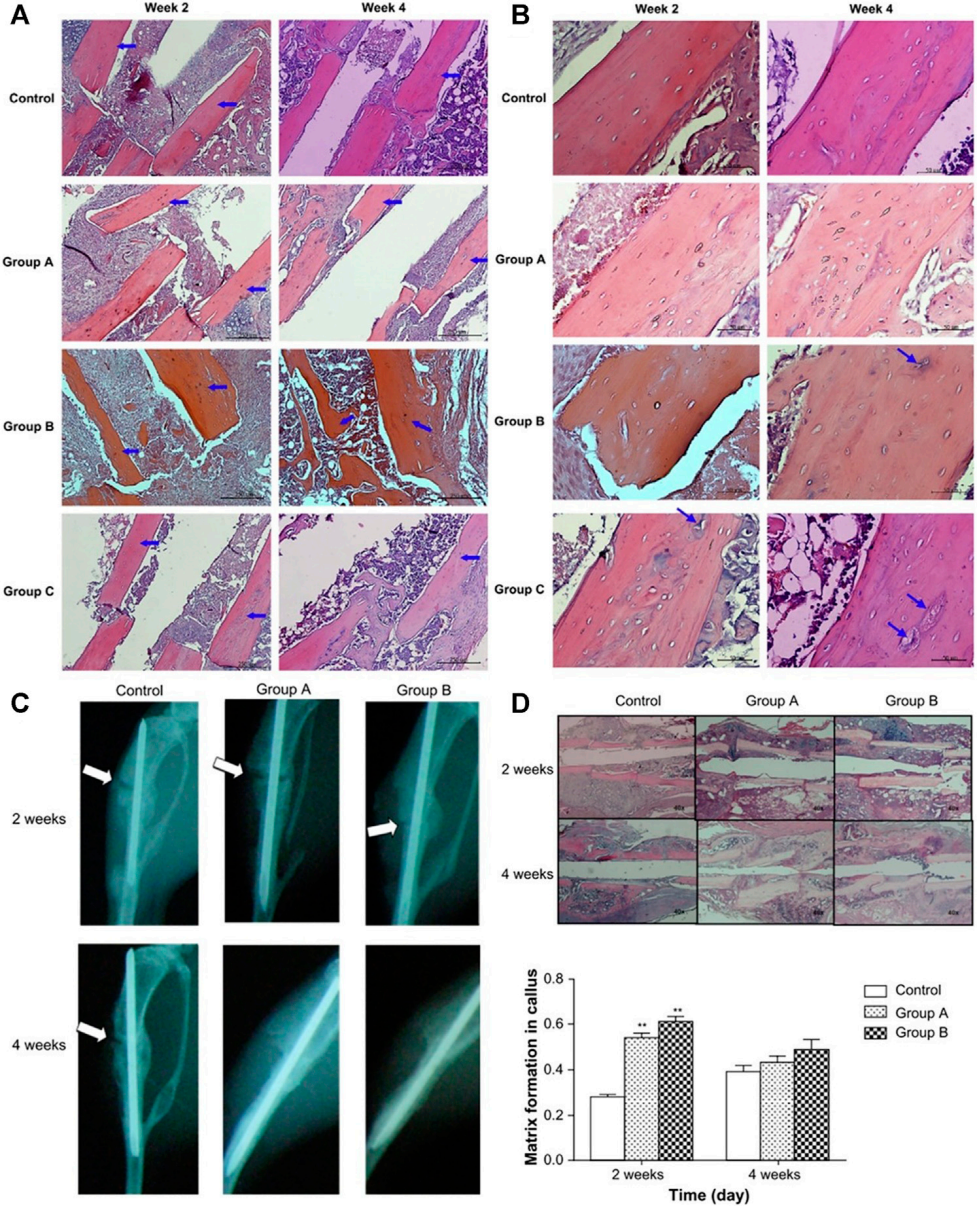
Combination of polymers and bioactive factors. Histological specimens from mice tibias were made after 2 and 4 weeks of implantation of different rhBMP-2 carriers (Group A, Group B, Group C) along with control. Original magnification is 400x for **(A)**. Original magnification is 100x for **(B)**. (Group A: 8 mg HAP and 2,500 ng rhBMP-2 within PLGA microspheres, Group B: 1,000 ng rhBMP-2 coating on PLGA microspheres, Group C: 2 mg HAP and 2000 ng rhBMP-2 coating on PLGA microspheres) Blue arrows identify lacunae. (Wang et al., 2009). **(C)** Radiography of mice tibias 2 and 4 weeks after implantation of SIM/PLGA/HAP. No implantation was implanted in the bone fracture of the control group. The meaning of the white arrow has been clarified. (group A, 3 mg SIM/PLGA/HAP; group B, 5 mg SIM/PLGA/HAP) (Tai et al., 2013). **(D)** Hematoxylin-eosin staining and quantification of matrix formation in the callus by Image-Pro Plus. *p* < 0.01 compared with control (Tai et al., 2013). Reproduced with permission (Wang et al., 2009). Copyright 2009, Elsevier Ltd. Reproduced with permission (Tai et al., 2013). Copyright 2013, Dove Medical Press Ltd.

Growth factors can accelerate the differentiation of stem cells into osteoblasts and have been widely used in the study of osteonecrosis (Zuo and Gong, 2012; Zhao et al., 2018b). Among them, the most well-known include bone morphogenetic protein (BMP), vascular endothelial growth factor (VEGF), and BFGF. These factors are often used to transform biological materials. BMP promotes the differentiation of mesenchymal stem cells into osteoblasts in the human body, and is also the main factor in inducing bone and cartilage formation in the body (Ngo et al., 2006; Zhao et al., 2019a). These growth factors are mostly protein structures. In the harsh microenvironment of osteonecrosis, they are susceptible to losing their activity. As a carrier, the polymer scaffold effectively preserves the biological activity of the growth factor, stabilizes the growth factor in the target area, and ensures its release is sustainable (Zhang et al., 2019a; Cui et al., 2020). In addition, BMP can stimulate the formation of new blood vessels (van der Bent et al., 2002). Wang et al. (2009) encapsulated BMP-2 in PLGA/hydroxyapatite (HAP) microspheres and found that the microspheres could release a sufficient therapeutic concentration of BMP-2, and the biological activity of BMP-2 was well maintained. The results showed that new bone tissue appeared in the area of osteonecrosis (Figure 10A). The addition of BMP therefore improves the osteogenesis of polymers.

Previous studies have shown that oral simvastatin (SIM) has the function of promoting new bone formation in the damaged bone tissue of the human body, but statins are easily degraded during the first liver metabolism (Mundy et al., 1999; Maeda et al., 2001; Song et al., 2003; Baek et al., 2005; Solomon et al., 2005). Tai et al. (2013) took advantage of the biodegradability of PLGA and encapsulated simvastatin in PLGA/HAP composite microspheres to obtain SIM/PLGA/HAP composite microspheres. The SIM/PLGA/HAP composite microspheres have a slow-release function, which effectively avoids rapid loss of simvastatin biological activity in the body. The SIM/PLGA/HAP microspheres were implanted into the osteonecrosis area in a mouse osteonecrosis model, and it was found that the SIM/PLGA/HAP microspheres promoted bone healing in mice and promoted the formation of new bone in the osteonecrosis area (Figure 10C). The investigators believed that the SIM/PLGA/HAP system will have a promising future in the treatment of osteonecrosis. Their study also confirmed that statins could promote the repair of osteonecrosis, because the combination of statins and polymers endows the polymers with osteogenic properties.

### Combination of Polymer and Bioactive Substance With Angiogenesis Function

**FIGURE 11:**
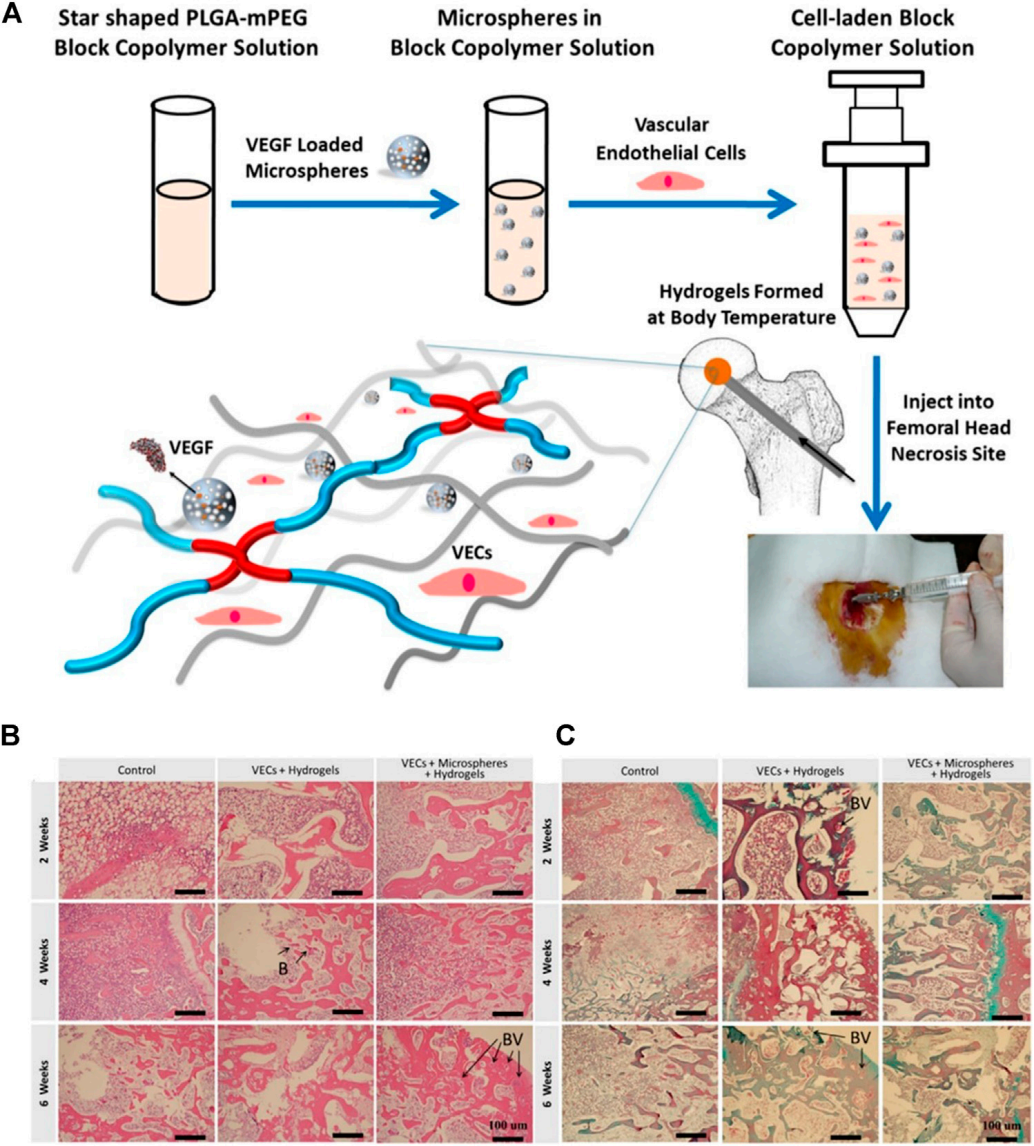
Polymer scaffold carrying VEGF is beneficial to vascular regeneration during osteonecrosis. **(A)** Schematic illustration for preparation of injectable hydrogel composite with VEGF loaded microspheres and vascular endothelial cells. Representative histological evaluation of cross sections retrieved at week 2, 4 and 6 after implantation of hydrogels composited with VEGF loaded microspheres. VEGF refers to vascular endothelial growth factor, VECs refers to vascular endothelial cells, **(B)** refers to blood cells, BV refers to blood vessels. The black arrow refers to the position of **(B)** or BV in the figures. Reproduced with permission (Chen et al., 2018). Copyright 2018, Elsevier Ltd.

Sufficient angiogenesis is necessary for the long-term survival of osteoblasts in the necrotic area (Koike et al., 2004; Zhang et al., 2018b). VEGF is considered to be a key regulator of angiogenesis in the process of bone repair (Ferrara et al., 2003). VEGF stimulates the reconstitution of blood supply at the site of necrosis, can induce the adhesion and proliferation of osteoblasts, and also promote the formation of new bone (Bai et al., 2014; Zhang et al., 2016). Chen et al. (2018) loaded VEGF into PLGA copolymer microspheres. VEGF-loaded PLGA copolymer microspheres and vascular endothelial cells (VECs) were composited into to form hydrogels (Figure 11A). Then they implanted the hydrogels into the rabbit model of femoral head necrosis. The results showed that the continuous release of VEGF caused a constant increase in new blood vessels, indicating that the polymer carrying VEGF is beneficial to vascular regeneration during osteonecrosis (Figures 1C, 11B).

BFGF, because of its effect on gene expression and angiogenesis, is considered to be a critical factor in the process of bone repair (Hu et al., 2015). used BFGF as a cytokine to transfect BMMSC, co-cultured it with XACB to construct functionalized XACB, and transplanted it into a rabbit model to repair osteonecrosis. The results showed that this method could effectively promote angiogenesis in the avascular necrosis area and significantly improve repair in osteonecrosis.

Some small molecule drugs also promote angiogenesis. The active ingredient of ginseng, ginsenoside Rg1, regulates angiogenesis and stimulates blood vessel formation by up-regulating the expression of nitric oxide and VEGF (Yue et al., 2007). Chang et al. (2010) added Rg1 to a CPC/PPF composite bone cement material, and the results showed that more new blood vessels were formed in the osteonecrotic area when Rg1 bone cement was added (Figure 9B). The addition of these bioactive factors with angiogenic activity promotes the angiogenic properties of polymers in osteonecrosis treatment.

### Combination of Polymer and Bioactive Substance With Dual Functions of Osteogenesis and Angiogenesis

Osteogenesis and angiogenesis are the most critical functions of functionalized polymers, and most functionalized polymer bone substitutes are constructed with this goal. The combination of osteogenesis and angiogenesis is more advantageous in osteonecrosis therapy than either process alone. Osteogenesis and angiogenesis are complementary: the new blood vessels provide oxygen and nutrients for the new bone tissue and remove the metabolic waste from the new bone tissue. The pore structure of new bone tissue provides space and mechanical support for new blood vessels.

Many small molecule drugs also promote bone formation and angiogenesis and are cheaper and more stable in the human body than stem cells and growth factors (Lo et al., 2012; Laurencin et al., 2014). Icariin is the active extract of epimedium, which can promote the activity and mineralization of osteoblasts and the formation of capillaries (Yue et al., 2007; Yao et al., 2012; Song et al., 2013; Tang et al., 2015b; Qin et al., 2015). Qin et al. (2015) added icaritin to a PTI composite scaffold. Compared with the simple PLGA/TCP (PT) group, *in vivo* experiments confirmed that the PTI scaffold group had the lowest incidence of femoral head collapse, better cartilage preservation, and more new bone formation in the bone tunnel. Deferoxamine is an iron chelator that stimulates the expression of angiogenic genes and promotes osteogenic differentiation of osteoblasts (Yan et al., 2019). Li et al. (2015) loaded deferoxamine on a gelatin sponge to enhance bone regeneration in patients with osteonecrosis. However, the results showed that the mechanical properties of the composite gel sponge and the sustained release of drugs were insufficient in the repair of osteonecrosis.

Some metal ions (such as strontium (Sr), Mg, and Li) also have dual functions of osteogenesis and angiogenesis, and they are often used in osteonecrosis research. Strontium has a chemical structure similar to calcium and stimulates bone formation, inhibits osteoclast differentiation, and promotes angiogenesis (Bonnelye et al., 2008; Zhao et al., 2018a). Kang et al. (2015) found that a strontium-doped calcium polyphosphate scaffold promotes angiogenesis and osteogenesis in osteonecrosis treatment. As an implantable metal material, Mg has good mechanical properties and biodegradability and can promote bone growth and microvascular expansion. Lai et al. (2019) added Mg to the PT scaffold to make the PTM scaffold, which promoted both osteogenesis and angiogenesis and had the synergistic effect of enhancing the formation of new bone and enhancing the quality of new bone in the rabbit model of osteonecrosis. The PTM scaffold had stronger osteogenic and angiogenic properties than the PT scaffold. Because Li enhances bone formation, promotes vascularization, and inhibits fat production, it has potential value in repairing osteonecrosis (Tang et al., 2015a; Li et al., 2017). As mentioned above, Li et al. (2018a) added Li to the composite scaffold and determined that the composite scaffold has the ability to promote bone formation and vascularization.

Many studies have found that the osteogenesis and angiogenesis functions of various growth factors have a synergistic effect, and their combined application can achieve a better osteonecrosis repair effect than a single growth factor (Garcia et al., 2012; Rackwitz et al., 2012; Bai et al., 2014; Zhang et al., 2016; Peng and Wang, 2017; Zhu et al., 2020). Peng and Wang (2017) transfected BMP-2 and BFGF into BMMSC and then loaded the modified bone marrow mesenchymal stem cells onto DBM. Implanting it into the canine model of ONFH promoted the bone repair effect in the area of osteonecrosis. Zhang et al. (2016) co-loaded BMP and VEGF into PLGA/CPC microspheres. The composite microspheres showed good biocompatibility and promoted bone formation and angiogenesis in animal experiments. Compared with other treatments, more bone and angiogenesis can be seen around the composite microspheres loaded with BMP and VEGF.

**FIGURE 12:**
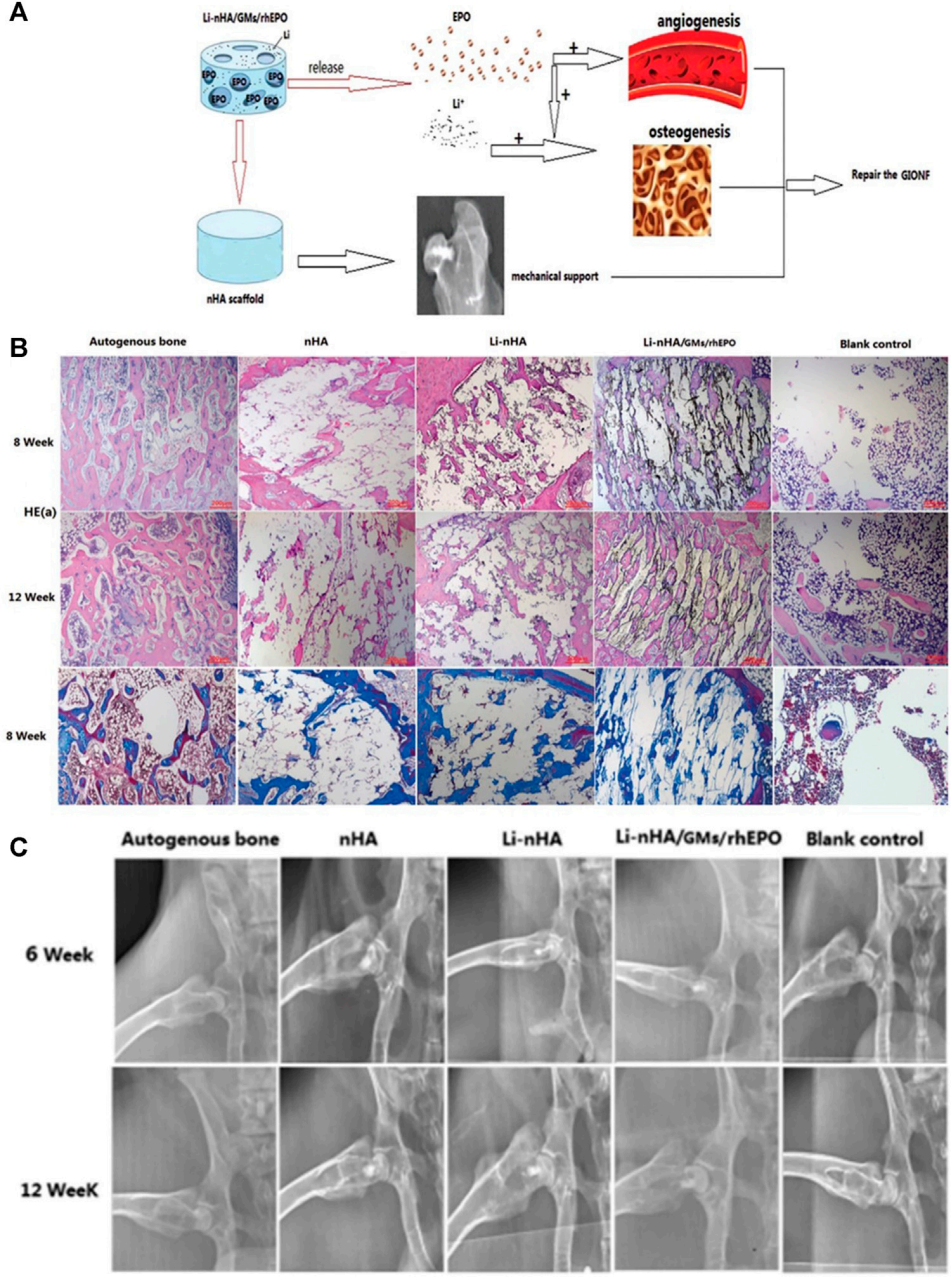
Combination of EPO, Li and polymers to treat osteonecrosis. **(A)** Schematic of the composite scaffold design. **(B)** HE staining and Masson staining showing the bone defect repair in the drilling channels. **(C)** X-radiographic examination showing the implants in the femoral head and the morphology of the femoral head. Reproduced with permission (Li et al., 2018a). Copyright 2018, The Royal Society of Chemistry 2018.

EPO is a pleiotropic cytokine that can enhance the function of VEGF and accelerate the differentiation of bone marrow mesenchymal stem cells into osteoblasts. Li et al. (2018a) combined EPO, gelatin, Li, and hydroxyapatite to make a composite scaffold (Figure 12A) and evaluated its mechanical properties, release properties, and *in vitro* biological activity. They implanted the scaffold into the femoral head of ONFH rabbits to evaluate the bone formation and angiogenesis ability of the stent *in vivo* and the effect in repairing bone defects. The results showed that the composite scaffold had good mechanical compressive strength. It could continuously release Li and EPO, enhance the formation of new bone and new blood vessel in ONFH rabbits, and had some effect in repairing femoral head necrosis (Figures 2C, 12B).

Many pathological changes occur during the development of osteonecrosis. It is challenging to achieve sufficient therapeutic effects with pure polymer materials. Therefore, functional polymer materials are being developed. The combination of polymers and biologically active substances achieves targeted therapy and maximizes the effectiveness of each, which is of great significance for the treatment of osteonecrosis.

## Conclusion and Future Perspectives

Osteonecrosis often affects the articular surface and is especially common in the femoral head. Osteonecrosis that has not been effectively treated will eventually cause the articular surface to collapse, leading to arthritis. The treatment of osteonecrosis has always attracted the attention of the medical community, and various methods have been explored to relieve and treat it. Treatment is mainly divided into two categories, surgical and nonsurgical treatments. At present, the primary clinical treatment is surgery. In the surgical treatment of osteonecrosis, joint replacement surgery is generally considered as a final intervention. Because the life of the artificial joint is limited, it may require multiple revision operations, which undoubtedly increase the pain and economic burden for the patient (Cao et al., 2016). Therefore, investigators continue to explore effective managements for osteonecrosis therapies. One therapy is core decompression combined with bone grafting, which can reduce intramedullary pressure in the necrotic area and trigger revascularization, bone formation, and remodeling by inducing local bone damage. Research on bone graft substitutes has become a significant field of study in osteonecrosis research. With the development of biotechnology and materials science, more potential biomaterials can be used to research osteonecrosis treatment.

A variety of organic and inorganic materials have been studied to treat osteonecrosis (Zhang et al., 2019a; Zhu et al., 2020). Because polymers have advantages over inorganic materials concerning biocompatibility, biodegradability, and mechanical properties, polymers have received more attention than inorganic materials. However, because of the harsh microenvironment of the area of osteonecrosis, pure polymers are not suitable for treating osteonecrosis. The combined application of polymers and various other substances harnesses the advantages of various substances with the strengths of polymers to meet a broader range of requirements in osteonecrosis research. The addition of various substances improves the biological activity and mechanical support performance of pure polymers. Various biologically active substances are added to polymers to produce functionalized polymers. Adding stem cells, growth factors, small molecule drugs, and metal ions to the polymer bone substitute materials endow the polymer with osteogenic and vascular properties that are beneficial in repairing osteonecrosis. Research on functionalized polymer bone substitute materials has become a developing trend.

The relevant experiments mentioned in this review were all carried out in animal models. Animal models of osteonecrosis cannot fully simulate the process of human osteonecrosis. The vast majority of research is limited to animal experiments, and research results cannot soon be translated into clinical practice. Therefore, the development of more ideal animal models for osteonecrosis research is necessary for the future.

The current composites cannot achieve the optimal coordination of various properties, such as the mutual influence between porosity, degradability, and mechanical properties (Zhu et al., 2020). The combined ratio of various materials also affects the properties of composite materials. Future research should mainly focus on improving existing materials and the development of new materials to enhance the properties of various materials.

The reconstruction of human bone tissue is a complex process that involves the synergy of many tissues, cells, and biological factors. We believe that the next step of functionalizing polymer materials should be to add more cells, growth factors, drugs, and other biologically active materials rather than just a few biologically active materials. The interaction between various biologically active factors related to osteonecrosis should also be studied more.

In summary, the creation of various functionalized polymer biomaterials may improve the treatment of osteonecrosis. We believe that future scientific and technological innovations and research can eventually result in significantly better treatment of osteonecrosis.
